# Knowledge, Attitude, and Practice Related to Contraception Use Among Childbearing Women in the Al-Ahsa Region, Saudi Arabia

**DOI:** 10.7759/cureus.88005

**Published:** 2025-07-15

**Authors:** Haidar N Alhassan, Mohammed AlKhamis, Mohammed N Alkhudhair

**Affiliations:** 1 Department of Family Medicine, Al-Ahsa Health Cluster, Al-Ahsa, SAU

**Keywords:** attitude, contraception, family planning, knowledge, practice, saudi arabia

## Abstract

Introduction: Family planning is crucial to manage population growth, reduce resource pressure, and enhance the quality of life while also promoting gender equality and improving educational options. While Saudi Arabia’s birth rate has declined, it remains high compared to high-income countries. Effective contraception reduces unintended pregnancies and associated maternal health risks. The study aimed to assess the knowledge, attitudes, and practices related to contraceptive methods among childbearing women in the Al-Ahsa region, Saudi Arabia, in 2025.

Methods: This descriptive cross-sectional community-based study was conducted in 2025 among childbearing women residing in the Al-Ahsa region, Saudi Arabia. A convenience sampling yielded 392 participants. The data were collected using a pretested, validated online questionnaire and analyzed by Statistical Package for Social Sciences software for Windows, version 27 (IBM Corp., Armonk, NY). Both descriptive and inferential statistics were applied, with a p value of ≤0.05 considered statistically significant.

Results: This study included 392 Saudi women with a mean age of 33.52 (±8.94) years. Nearly half of them (49.7%) held a bachelor's degree, and 33.4% were employed. Most of the participants (90.3%) had heard of contraception, with 50.3% identifying it as a method of family planning and 45.2% as a way to prevent unwanted pregnancy. Regarding awareness of contraceptive methods, oral contraceptive pills (OCPs; 82.9%) and male condoms (70.7%) were the most recognized. Regarding attitude toward contraception usage, more than half (52.3%) believed that contraceptives are harmful, while 22.2% viewed contraception as useful. More than one-third of responders (37.5%) reported using contraception, with the most common reason being prevention of unplanned pregnancy (33.4%). OCPs and male condoms were the most used methods (12.5% and 12.8%, respectively). Contraceptive use was more prevalent among women aged 26-35 years, those married for three to five years, and those with multiple pregnancies or children.

Conclusion: Although awareness of contraception was high among participants, misconceptions and negative perceptions regarding its safety persist. To enhance contraception use, it is crucial to address prevailing negative attitudes through targeted public health campaigns that debunk myths and promote the benefits of contraception.

## Introduction

The world population in 2024 was eight billion, and it is still increasing [1-3]. Regulating fertility will help limit population growth, which will result in a decrease in the burden on resources of many developing countries; therefore, many developing countries consider limiting population growth a fundamental strategy to improve quality of life and standard of living, especially with the availability of effective contraception methods [4,5]. Even though the birth rate in the Kingdom of Saudi Arabia has decreased recently, it is still high compared with industrialized countries [5-7]. Family planning through contraceptive use plays a key role in reducing pregnancy rates [8]. Using contraceptive methods will lead to a reduction in the number of unintended pregnancies, which, in turn, reduces maternal morbidity and mortality, providing a substantial public health benefit [8,9]. There are multiple health benefits as well as health-related risks from using contraceptive methods, highlighting the importance of contraceptive counseling to support safe and effective use [8,10-13].

In 2022, the global contraceptive use was 65% for any method and 58.7% for modern methods in both married and union women [14]. Family planning has significantly increased over the past decades; the number of women who desire it increased from 900 million in 2000 to 1.1 billion in 2021 [15]. According to the United Nations, in 2023, 21% of women aged 15-49 in the Kingdom of Saudi Arabia used contraceptives, regardless of the method [14].

Literature reveals varying levels of knowledge, positive attitudes, and common practices regarding contraception among women in different regions in Saudi Arabia. Despite the general high awareness of contraception, there are notable differences in detailed knowledge and factors influencing its usage [16-18].

There is limited research on contraceptive knowledge, attitudes, and practices (KAP) among childbearing women in the Al-Ahsa region, Saudi Arabia. While studies exploring contraceptive KAP have been conducted in other regions of the Kingdom, the unique sociocultural and socioeconomic characteristics of Al-Ahsa necessitate localized data collection. These localized KAP data are vital for developing effective, culturally sensitive public health strategies to improve contraceptive use and reproductive health. This study aims to fill this critical knowledge gap, providing region-specific insights that will contribute to a more comprehensive national understanding of contraceptive behavior. This, in turn, will inform evidence-based policy development and resource allocation to enhance maternal and child health outcomes throughout Saudi Arabia. Subsequently, the aim of this study is to assess the knowledge, attitudes, and practices relating to contraceptive methods among women in the Al-Ahsa region, Saudi Arabia, in 2025.

## Materials and methods

Study design

A descriptive, cross-sectional, community-based study was conducted in 2025 among childbearing women in the Al-Ahsa region, Saudi Arabia. By using Open Epi version 3.0 (www.openepi.com; Bill and Melinda Gates Foundation, Atlanta, GA), the population size of Saudi women living in Al-Ahsa is about 416,844, according to the General Authority for the Statistics of the Kingdom of Saudi Arabia [19]. Assuming 50% of women knew about family planning, the minimum sample size to achieve the confidence interval level of 95% and 5% margin of error was 385. After accounting for 10% and excluding some responses, the final sample included 392 participants via convenience sampling.

Study instrument

Data were collected using a pretested, online questionnaire that had been previously used and validated in a published work in another similar study [17]. The questionnaire included questions about sociodemographic characteristics, knowledge, attitudes, and practices regarding contraceptive method usage. The study utilized the Arabic version of the questionnaire after obtaining consent from its authors. The questionnaire was designed for confidentiality and anonymity, so no personally identifiable information was collected.

Data analysis

Data were collected online and exported into an Excel sheet (Microsoft Corporation, Redmond, WA), coded, and then analyzed using the Statistical Package for Social Sciences software for Windows, version 27 (IBM Corp., Armonk, NY). Continuous variables were presented as means and standard deviations, and categorical variables were presented as frequencies and percentages. The chi-square test was used to determine the relationship between contraceptive use and sociodemographic characteristics, with a p value of ≤0.05 considered statistically significant.

Ethical statement

This study was ethically approved by the Institutional Review Board of the Al-Ahsa cluster (R441024-EP-2024). Before participation, all subjects provided electronic informed consent. This process included a comprehensive explanation of the study's objectives, as well as an explicit assurance of their right to withdraw from the study at any point without consequence. Consent was obtained electronically to document their agreement to submit their responses.

## Results

In a total of 392 responses, the majority of women were married (83.4%), with a mean age of 33.52 (±8.94) years, and the largest age group was between 26 and 35 years (39.8%). Nearly half of the participants (49.7%) held a bachelor's degree, and 33.4% were employed, while 45.4% were housewives. More than half (57.4%) of participants reported a monthly income of less than 5,000 SAR. More than half of the participants had been married for over five years (61.5%). Regarding fertility, 46.9% had two to four children, while 34.4% reported a previous history of abortion (Table 1).

**Table 1:** Socioeconomic characteristics (n = 392) The age, mean (±SD), of all women is 33.52 (±8.935) SD: standard deviation; SAR: Saudi Riyal

Socioeconomic characteristics		Frequency	Percentage
Marital status	Single	45	11.5%
	Married	327	83.4%
	Separated	20	5.1%
Age	18-25 years	82	20.9%
	26-35 years	156	39.8%
	Above 35 years	154	39.3%
Education level	Primary school	21	5.4%
	Intermediate	22	5.6%
	Secondary	77	19.6%
	Diploma	38	9.7%
	Bachelor's	195	49.7%
	Master's or doctorate	35	8.9%
	Illiteracy	4	1.0%
Job	Employed	131	33.4%
	Student	61	15.6%
	Housewife	178	45.4%
	Retired	3	0.8%
	Unemployed	19	4.8%
Monthly income	<5,000 SAR	225	57.4%
	5,000-10,000 SAR	72	18.4%
	11,000-15,000 SAR	35	8.9%
	>15,000 SAR	60	15.3%
How long have you been married?	<1 year	10	2.6%
	1-2 years	30	7.7%
	3-5 years	56	14.3%
	>5 years	241	61.5%
	Not married	55	14.0%
Number of children	No children	77	19.6%
	One child	70	17.9%
	Two to four children	184	46.9%
	Five or more children	61	15.6%
Previous history of abortion	Yes	135	34.4%
	No	257	65.6%

The majority of women (90.3%) had heard of contraception. When asked about its meaning, 50.3% identified it as a method of family planning, 45.2% as a method to prevent unwanted pregnancy, and 8.2% believed it protects against sexually transmitted diseases. Regarding awareness of contraceptive methods, oral contraceptive pills (OCPs; 82.9%) and male condoms (70.7%) were the most recognized, followed by intrauterine devices (IUDs; 60.7%) and contraceptive implants (58.7%). Less commonly known methods included cervical caps (15.6%), contraceptive rings (22.7%), and spermicides (14.3%). Despite this, 40.6% of participants expressed a need for more education on family planning (Table 2).

**Table 2:** Knowledge of responders regarding contraception (n = 392)

Knowledge regarding contraception		Frequency	Percentage
Have you ever heard of contraception?	Yes	354	90.3%
	No	38	9.7%
According to your knowledge, what does contraception mean?	Method of family planning	197	50.3%
	Method to prevent unwanted pregnancy	177	45.2%
	Could protect against sexual disease	32	8.2%
	All of the above	103	26.3%
	Do not know	33	8.4%
What type of contraception do you know of?	Male condom	277	70.7%
	Oral contraceptive pills	325	82.9%
	Emergency contraception pill	154	39.3%
	Intrauterine device	238	60.7%
	Contraceptive implant	230	58.7%
	Contraceptive patch	166	42.3%
	Sterilization (vasectomy or fallopian ligation)	131	33.4%
	Contraceptive injection	132	33.7%
	Cervical cap	61	15.6%
	Contraceptive ring	89	22.7%
	Female condom	93	23.7%
	Spermicides	56	14.3%
	Do not know	34	8.7%
Do you need more education regarding family planning?	Yes	159	40.6%
	No	233	59.4%

**Figure 1:**
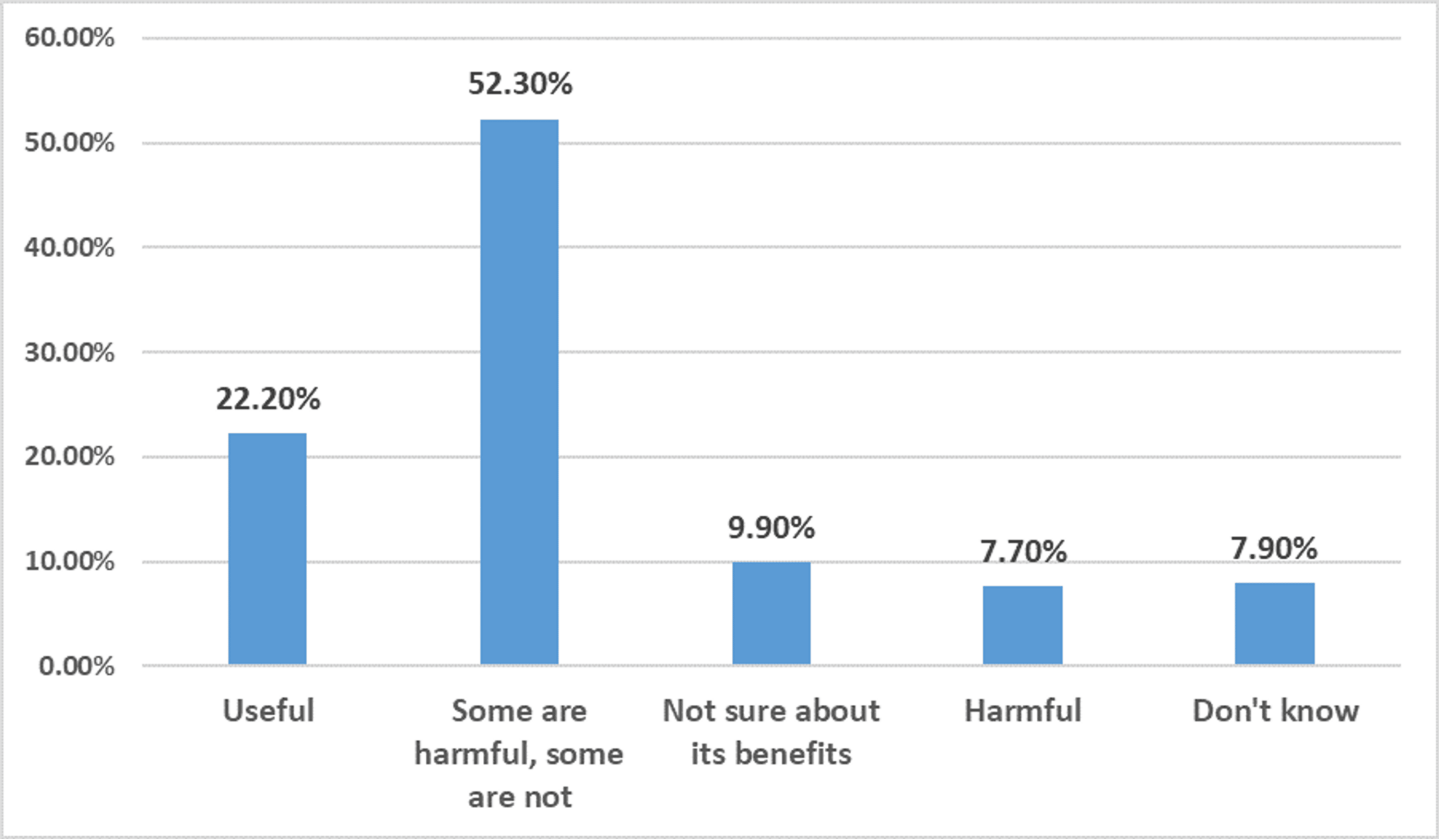
Attitude of responders regarding contraception usage (n = 392)

Regarding attitude toward contraception usage, the majority (52.3%) believed that "some contraceptives are harmful while others are not," reflecting a mixed perception of safety. A smaller percent (22.2%) viewed contraception as useful, while 9.9% were unsure about its benefits. Additionally, 7.7% considered contraceptive methods harmful, and 7.9% reported that they did not know (Figure 1).

The vast majority (93.1%) of women reported preferring to space births, indicating a strong inclination toward allowing time intervals between pregnancies, while only 6.9% did not prefer spacing. Regarding the preferred time between births, the majority (56.1%) favored a three-to-four-year interval, followed by 19.1% who preferred more than four years and 15.6% who preferred one to two years (Table 3).

**Table 3:** Responder's perception of regarding birth spacing, practice of contraception usage, and reasons of nonuse (n = 392) ^†^This question has multiple answers OCPs: oral contraceptive pills; IUD: intrauterine device

Variables		Frequency	Percentage
Do you prefer spacing births, meaning leaving a certain time interval between the two times you have children?	Yes	365	93.1%
	No	27	6.9%
What is your favorite time period?	<1 year	9	2.3%
	1-2 years	61	15.6%
	3-4 years	220	56.1%
	>4 years	75	19.1%
Do you use contraception?	Yes	147	37.5%
	No	245	62.5%
What is the reason for your use of contraception?	Prevent unplanned pregnancy	131	33.4%
	Regulate menstrual cycle	8	2.0%
	Prevent pregnancy due to health problems	5	1.3%
	Other	12	3.1%
The type of contraception used^†^	OCPs	49	12.5%
	Male condom	50	12.8%
	IUD	13	3.3%
	Natural family planning	38	9.7%
	Contraceptive implant	11	2.8%
	Cervical cap	1	0.3%
	Withdrawal method	10	2.6%
Have you ever gotten pregnant while using contraception?	Yes	28	7.1%
	No	364	92.9%
What method of contraception did you use?	Natural method	9	2.3%
	Contraceptive pills	11	2.8%
	Withdrawal method	9	2.3%
	Male condom	6	1.5%
	Contraceptive implant	2	0.5%
Did you experience any side effects while using birth control?	Yes	52	13.3%
	No	340	86.7%
What side effects did you experience?	Bleeding	17	4.3%
	Frequent nausea and vomiting	12	3.1%
	Breast pain	10	2.6%
	Mood changes	23	5.9%
	Weight gain	22	5.6%
	Hair loss	17	4.3%
	Chronic headaches	14	3.6%
	Current pregnancy	35	8.9%
	Other symptoms	7	1.8%
Why are you not using birth control?	Using the natural method	39	9.9%
	Fear of side effects	28	7.1%
	Mutual agreement between the couple	32	8.2%
	I have a desire to have children	51	13.0%
	For medical reasons or health issues	20	5.1%
	Breastfeeding	13	3.3%
	No support from my husband	5	1.3%
	Unmarried	48	12.2%

Regarding the practices of contraception use, more than one-third of responders (37.5%) reported using contraception, with the most common reason being prevention of unplanned pregnancy (33.4%). OCPs and male condoms were the most used methods (12.5% and 12.8%, respectively), followed by natural family planning (9.7%) and IUDs (3.3%). In addition, a small proportion of responders (7.1%) reported pregnancy while using contraception, most commonly with natural methods or withdrawal. Among contraception users, 13.3% experienced side effects. The most reported were mood changes (5.9%), weight gain (5.6%), and bleeding (4.3%). Despite this, 86.7% did not report any side effects (Table 3).

Regarding reasons for nonuse, 62.5% were not using contraception. The primary reasons included desire for children (13%), being unmarried (12.2%), using natural methods (9.9%), mutual agreement with the partner (8.2%), and fear of side effects (7.1%). Less commonly cited reasons included health issues, breastfeeding, or lack of partner support (Table 3).

The chi-square analysis reveals there were significant associations with age, marital status, duration of marriage, times of previous pregnancy history, and number of children. Specifically, women aged 26-35, those married for three to five years, multigravida, and those who have multiple children are more likely to use contraception. Conversely, education level, occupation, monthly income, and previous history of abortion had no statistically significant association with contraception usage (Table 4).

**Table 4:** Chi-square test for association between demographic variations and contraception usage ^*^A statistically significant finding with a p value of less than 0.05 SAR: Saudi Riyal

Variables		Do you use contraception?		p value
		Yes	No	
Age categories	18-25 years	22 (26.8%)	60 (73.2%)	0.020^*^
	26-35 years	70 (44.9%)	86 (55.1%)	
	More than 35 years	55 (35.7%)	99 (64.3%)	
Marital status	Single	1 (2.2%)	44 (97.8%)	<0.001^*^
	Married	145 (44.3%)	182 (55.7%)	
	Separated	1 (5.0%)	19 (95.0%)	
Education level	Primary school	6 (28.6%)	15 (71.4%)	0.302
	Intermediate	4 (18.2%)	18 (81.8%)	
	Secondary	31 (40.3%)	46 (59.7%)	
	Diploma	12 (31.6%)	26 (68.4%)	
	Bachelor's	75 (38.5%)	120 (61.5%)	
	Master's or doctorate	17 (48.6%)	18 (51.4%)	
	Illiteracy	2 (50.0%)	2 (50.0%)	
Occupation	Employed	53 (40.5%)	78 (59.5%)	0.427
	Student	21 (34.4%)	40 (65.6%)	
	Housewife	64 (36.0%)	114 (64.0%)	
	Retired	0 (0.0%)	3 (100.0%)	
	Unemployed	9 (47.4%)	10 (52.6%)	
Monthly income	<5,000 SAR	81 (36.0%)	144 (64.0%)	0.405
	5,000-10,000 SAR	27 (37.5%)	45 (62.5%)	
	11,000-15,000 SAR	11 (31.4%)	24 (68.6%)	
	>15,000 SAR	28 (46.7%)	32 (53.3%)	
How long have you been married?	<1 year	2 (20.0%)	8 (80.0%)	<0.001^*^
	1-2 years	11 (36.7%)	19 (63.3%)	
	3-5 years	28 (50.0%)	28 (50.0%)	
	>5 years	104 (43.2%)	137 (56.8%)	
	Not married	2 (3.6%)	53 (96.4%)	
How many times have you been pregnant?	Once	23 (41.1%)	33 (58.9%)	<0.001^*^
	Twice	36 (48.6%)	38 (51.4%)	
	Three times	23 (39.7%)	35 (60.3%)	
	Four times	21 (38.9%)	33 (61.1%)	
	Five times or more	40 (45.5%)	48 (54.5%)	
	Not occurred	4 (6.5%)	58 (93.5%)	
Number of children	No children	3 (3.9%)	74 (96.1%)	<0.001^*^
	One child	31 (44.3%)	39 (55.7%)	
	2-4 children	83 (45.1%)	101 (54.9%)	
	5 or more children	30 (49.2%)	31 (50.8%)	
Previous history of abortion	Yes	56 (41.5%)	79 (58.5%)	0.238
	No	91 (35.4%)	166 (64.6%)	

## Discussion

Childbirth is the primary cause of mortality among women of reproductive age, with one in seven women in this demographic dying because of complications related to childbirth [20]. In our study, 90% of women know what contraception means, 50% understand that it serves as a method of family planning, 45% recognize it to prevent unwanted pregnancies, and 8% identify it as a method for protection against sexually transmitted diseases. A recent study conducted in Abha, KSA, indicated that 80.6% of their participants were aware of family planning, and among them, 68.1% were able to define family planning accurately [21].

Regarding family planning methods, OCPs were recognized by 82.9% of the population as the most familiar method, followed by male condoms at 70.7%, IUDs at 60.7%, and contraceptive implants at 58.7%, while other methods were mentioned by only a few participants. These findings are similar to a recent study in the Makkah region, which showed that women of childbearing age recognized OCPs, IUDs, and male condoms the most, with rates of 97%, 92%, and 88%, respectively. Additionally, another study conducted among Saudi men showed that OCP, condoms, and the withdrawal method were the most recognized methods [22]. Furthermore, regarding permanent contraception such as sterilization (vasectomy or fallopian ligation), our participants showed fair knowledge, with one-third (33.4%) indicating that they are aware of it. This percentage was higher than another Saudi study that indicated that a few women were aware of fallopian ligation (15.6%) and male sterilization (11.2%) [23].

This study revealed that approximately one-third of women (37%) use contraception (either traditional or modern methods). Similarly, Saudi studies conducted in the Abha, Makkah, and Al-Qassim regions revealed that 29%, 37%, and 45% of their women used contraception, respectively [7,17,21]. Inconsistently, this rate of contraception usage was lower than the findings from various studies conducted in different areas, such as Asser-KSA (75%), Syria (47%), and Egypt (60%) [24,25]. The reduced rate of contraception usage in this study may be attributed to the selection criteria, which include women with different education levels and age groups, as well as unmarried women.

The current study revealed that the male condom was the major method used by 12.8%, followed by OCPs and natural family planning methods by 12.5% and 9.7%, respectively. Conversely, other methods like IUDs, contraceptive implants, and cervical caps had lower usage rates. This may be due to inadequate promotion by medical professionals, restricted access, and patient discomfort with invasive procedures for limited usage of these methods. A study from Singapore mentioned that male condoms were the most widely known and practiced method among their population, followed by OCPs, which is consistent with our study [26]. Inconsistently, a systematic review study revealed that OCPs were the most common contraception used in Saudi Arabia, followed by IUD [27]. Unlike previous Saudi studies, the permanent methods, such as vasectomy or fallopian ligation, were not used among our population [7]. This variation may be attributed to the participants' traditions and Islamic culture, which accept only temporary delays in pregnancy while rejecting permanent sterilization. This result contrasts with global findings, whereas female sterilization was one of the most commonly utilized modern methods among married women [28].

The present study revealed that more than one-fifth of our population (22%) thought that contraception is useful, 52% considered some forms to be harmful, and 9.9% were not sure about its benefits, while 7.7% perceived it as harmful. Studies from the Makkah Region and Al-Medina City revealed that more than half of their participants agreed that some contraceptives are beneficial and some are not, which is consistent with our study [17,29]. Inconsistently, a study from Indonesia indicated that 79.12% of their participants concurred that the utilization of contraception is beneficial [30]. These findings demonstrate different degrees of trust and awareness, indicating the need for more effective education and communication about the safety and effectiveness of contraceptive choices.

Furthermore, the vast majority of the participants (93%) preferred spacing births, whereas the favorite time interval was three to four years for more than half of the participants (56%). These results are inconsistent with previous Saudi studies in Al-Qassim center and Dammam city that showed the preferred interval period was two to three years, while a study in Hail mentioned that the favorite interval was two to four years [7,31].

This study revealed that the vast majority of women who used contraception used it to decrease unwanted pregnancies. Instances of unwanted pregnancies influence breastfeeding practices [32]. Literature examining abortion as a method of fertility control has indicated that women primarily turn to abortion when family planning services are lacking or inadequate [33]. Lower rates of abortion and pregnancy contribute to decreased maternal mortality by minimizing the frequency of risk exposure, as well as decreasing stillbirths and improving child mortality rates.

Regarding barriers to using birth control methods, the primary reason cited was the desire to have children, accounting for 13%. This was followed by being currently unmarried in 12.2%, currently pregnant in 8.9%, mutual agreement between partners in 8%, fear of side effects in 7%, and medical reasons in 5%, while 9.9% of women believed that employing natural methods was beneficial for them. A study conducted in Sierra Leone mentioned that the primary reason for not using contraception was unwillingness to disclose at 52.6%, followed by a wish for a child at 19.2%, apprehension regarding side effects at 15.7%, being currently pregnant at 8.7%, and being against religious beliefs at 3.5% [34]. A study in Al-Qassim center, KSA, revealed that the children's blessing was the major reason for contraceptive refusal in 69.5%, followed by fear of health problems (19%) and fear of affecting marital life (11%) [7]. Another study conducted in Cameroon revealed that the main reasons for not using contraception included insufficient information (31.4%), perceived uselessness (31.4%), and concerns regarding side effects (14.3%) [35].

The current study demonstrated a significant association between age and contraception-using rate, whereas mothers aged 26-35 years and those aged more than 35 years had the highest rate. Al Sheeha mentioned that the contraception use rate was higher in groups over 30 years old, with a strong, significant association [7]. Inconsistently, a study in the Makkah region mentioned that the highest contraception use was presented among the 18-25 age group with an insignificant association [17]. Older mothers attributed their feeling that they have enough children and want to be more concerned with their study and work, unlike younger ones.

Moreover, the present study revealed that there was a significant correlation between the number of children and the contraception usage rate, in which the rate was increasing as the number of children increased. Combined with this rate, 40% of our population stated that they need more information regarding contraception. This indicates the need for areas for improvement in public awareness and reproductive health education in Al-Ahsa Governorate.

Strengths and limitations of the study

This study provides good insights about contraceptive KAP among women in the Al-Ahsa region of Saudi Arabia, supported by a reasonable sample size of 392. However, the cross-sectional design limits causal inferences, and the convenience sampling may introduce selection bias, hindering generalizability. Reliance on self-reported data and an online questionnaire raises concerns about recall bias, social desirability bias, and potential exclusion of women with limited internet access.

## Conclusions

This study demonstrates a good level of awareness regarding contraception; a notable finding was the persistence of negative perceptions about its safety among more than half of the participants. Despite these concerns, over a third of the respondents reported using contraception, primarily to prevent unplanned pregnancies, with OCPs and male condoms being the most popular methods. The study further concludes that contraceptive use is significantly associated with specific demographic factors, including being in the 26-35 age group, having been married for three to five years, and having experienced multiple pregnancies or having several children. To address these findings, the study strongly recommends targeted public health campaigns designed to directly counter myths and misconceptions about contraception, thereby promoting a more positive and accurate understanding of its benefits and safety. Furthermore, it suggests focusing educational efforts on specific demographic groups, such as younger married women and those who are considering or actively expanding their families, to provide tailored information on various contraceptive options suitable for different life stages. These recommendations aim to bridge the gap between awareness and positive attitudes, ultimately leading to improved contraceptive practices.
